# Bottom-Up and Top-Down Attention Impairment Induced by Long-Term Exposure to Noise in the Absence of Threshold Shifts

**DOI:** 10.3389/fneur.2022.836683

**Published:** 2022-03-01

**Authors:** Ying Wang, Xuan Huang, Jiajia Zhang, Shujian Huang, Jiping Wang, Yanmei Feng, Zhuang Jiang, Hui Wang, Shankai Yin

**Affiliations:** ^1^Department of Otolaryngology-Head and Neck Surgery, Shanghai Jiao Tong University Affiliated Sixth People's Hospital, Shanghai, China; ^2^Otolaryngology Institute of Shanghai Jiao Tong University, Shanghai, China; ^3^Shanghai Key Laboratory of Sleep Disordered Breathing, Shanghai, China; ^4^Department of Otolaryngology, The First Affiliated Hospital, College of Medicine, Zhejiang University, Hangzhou, China

**Keywords:** noise, attention function, P300, mismatch negativity, bottom-up, top-down

## Abstract

**Objective:**

We aimed to assess the effect of noise exposure on bottom-up and top-down attention functions in industrial workers based on behavioral and brain responses recorded by the multichannel electroencephalogram (EEG).

**Method:**

In this cross-sectional study, 563 shipyard noise-exposed workers with clinical normal hearing were recruited for cognitive testing. Personal cumulative noise exposure (CNE) was calculated with the long-term equivalent noise level and employment duration. The performance of cognitive tests was compared between the high CNE group (H-CNE, >92.2) and the low CNE group; additionally, brain responses were recorded with a 256-channel EEG from a subgroup of 20 noise-exposed (NG) workers, who were selected from the cohort with a pure tone threshold <25 dB HL from 0.25 to 16 kHz and 20 healthy controls matched for age, sex, and education. P300 and mismatch negativity (MMN) evoked by auditory stimuli were obtained to evaluate the top-down and bottom-up attention functions. The sources of P300 and MMN were investigated using GeoSource.

**Results:**

The total score of the cognitive test (24.55 ± 3.71 vs. 25.32 ± 2.62, *p* < 0.01) and the subscale of attention score (5.43 ± 1.02 vs. 5.62 ± 0.67, *p* < 0.001) were significantly lower in the H-CNE group than in the L-CNE group. The attention score has the fastest decline of all the cognitive domain dimensions (slope = −0.03 in individuals under 40 years old, *p* < 0.001; slope = −0.06 in individuals older than 40 years old, *p* < 0.001). When NG was compared with controls, the P300 amplitude was significantly decreased in NG at Cz (3.9 ± 2.1 vs. 6.7 ± 2.3 μV, *p* < 0.001). In addition, the latency of P300 (390.7 ± 12.1 vs. 369.4 ± 7.5 ms, *p* < 0.001) and MMN (172.8 ± 15.5 vs. 157.8 ± 10.5 ms, *p* < 0.01) was significantly prolonged in NG compared with controls. The source for MMN for controls was in the left BA11, whereas the noise exposure group's source was lateralized to the BA20.

**Conclusion:**

Long-term exposure to noise deteriorated the bottom-up and top-down attention functions even in the absence of threshold shifts, as evidenced by behavioral and brain responses.

## Introduction

Noise is one of the most common types of pollution in both occupational and non-occupational environments (1). Long-term noise exposure that exceeds certain levels can harm the auditory system, resulting in progressive hearing loss and an increase in hearing sensitivity threshold (2, 3). Meanwhile, evidence of the non-auditory effects related to noise exposure is growing (4, 5), such as, annoyance (6), disturbed sleep (7), cardiovascular disease (8), and anxiety (9). In addition to these effects, noise exposure affects a variety of cognitive processes, such as reaction time, memory, perception, and attention (10). Human error and, in some cases, increased accidents may result from the alteration of attention performance (11). A previous study demonstrated that noise exposure could impair performance on the focused attention task (12), while some studies found that noise could increase arousal levels and accuracy in computerized attention tests (13). The effect of noise exposure on attention performance remain rather inconclusive (14, 15).

One of the influential parameters in the effect of noise on attention performance could be noise characteristics. Jafari et al. (10) discovered the decreased attention in low-frequency noise-exposed subjects (16) and a significant reduction of visual and auditory attention when noise intensity was at 95 dBA level. Smith and Miles (17) found that subjects who were exposed to noise for 5 h made more errors than those who were exposed for 2 h in a reaction time task. Pawlaczyk-Łuszczyńska et al. (18) discovered that the low-frequency noise might affect the concentration and attention function. Furthermore, exposure duration, intensity, education years, gender, age, hearing level, and even basic diseases could all be influential parameters regarding the effect of noise on attention performance and might lead to these apparently contradictory results.

Attention is not a monolithic process, and two types of attention are commonly distinguished: top-down and bottom-up attention (19, 20). The voluntary allocation of attention to certain features or objects is referred to as top-down attention (21). Attention, on the other hand, is not only voluntarily directed. Salient stimuli can attract attention, even though the subject has no intention of focusing on these stimuli (22). Bottom-up attention refers to solely being guided by externally driven factors to stimuli (22). The attention process can be modulated by “top-down” specific task goals and expectations as well as “bottom-up” external-driving factors (23). “Bottom-up” attention plays a critical role during auditory processing in noisy environments (24), which is capable of tracking certain auditory stimuli in noisy environments without paying attention voluntarily to the auditory modality. In tasks with several components, noise may cause an increase in concentration on the dominant or high-probability component at the expense of other features (12). However, there is still a scarcity of solid evidence from people who have documented the effects of noise exposure on top-down and bottom-up attention performance.

In this study, we aimed to evaluate the effect of noise exposure on bottom-up and top-down attention functions in industrial workers in the absence of peripheral hearing loss based on behavioral and brain responses recorded by the multichannel electroencephalogram (EEG). First, we utilized the Montreal Cognitive Assessment (MoCA) cognitive test to assess the cognitive performance, particularly attention, in a large cohort of shipyard workers with long-term noise exposure. In addition, we measured the P300 and the mismatch negativity (MMN), which reflect the brain's sound encoding, in a subgroup of 20 noise-exposed workers with pure tone thresholds <25 dB HL from 0.25 to 16 kHz, selected from the cohort and 20 healthy controls matched for age, gender, and education; their hearing functions were further evaluated by a comprehensive test battery containing both subjective and objective measures (25).

## Methods

### Participants and Study Design

**Figure 1:**
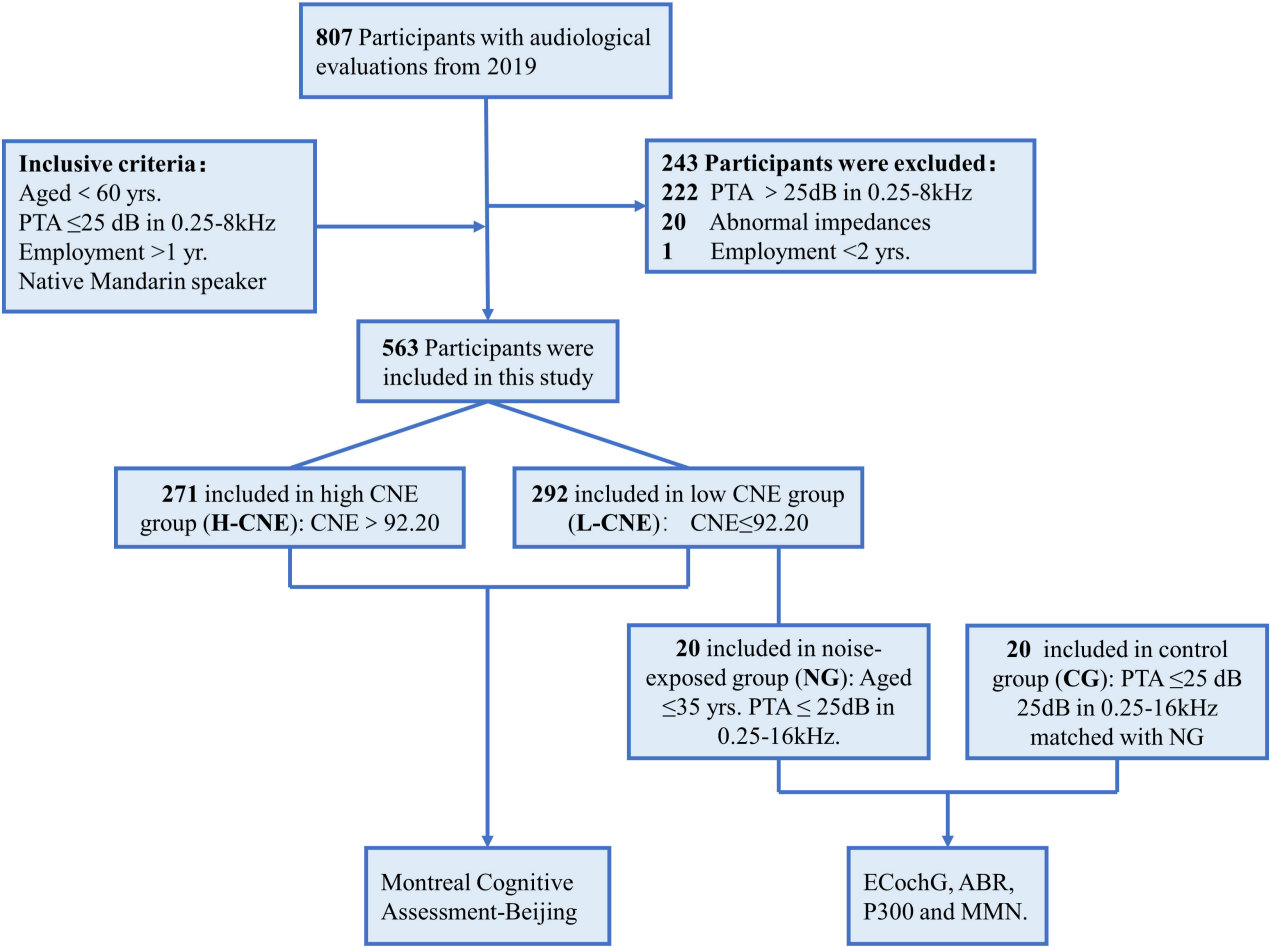
The flowchart illustrates the study design and participants.

A large-scale epidemiological survey was conducted from June to July 2019 (25). A questionnaire was used to collect the cross-sectional physical examination data from 807 sanding, welding, metal, and cutting workers, such as demographics, noise exposure duration, type of work, history of major diseases, including genetic and drug-related hearing loss, diabetes, hypertension, smoking, and alcohol consumption, and use of hearing protection devices. Audiologic evaluations and personal cumulative noise exposure (CNE) estimates were conducted, as described in our previous study (25). By the median (92.2 dBA-year) of CNE, all participants were divided into two groups: high CNE (H-CNE) and low CNE (L-CNE). Then, recruited participants completed cognitive tests to assess the cognitive function by professional physicals (26). The procedures and criteria for participant inclusion and exclusion are outlined in Figure 1. Inclusion criteria include: (1) age <50 years; (2) air conduction thresholds <25 dB HL at 0.25–8 kHz in bilateral ear; (3) employment duration > 2 years; (4) right-handed; and (5) native Mandarin speaker. Exclusion criteria include abnormal tympanograms, a history of otological diseases, or reading or language difficulties.

Furthermore, 20 participants were selected at random from L-CNE group as the noise-exposed group (NG) based on the following criteria: (1) under the age of 40 years; (2) pure-tone average (PTA) <25 dB hearing level at any frequency between 0.25 and 16 kHz; (3) right-handedness; and (4) native Mandarin speakers. The NG group underwent more extensive auditory processing tests, such as an electrocochleogram (ECochG) and auditory brainstem responses (ABR). A control group (CG) of 20 healthy subjects without a history of occupational noise exposure was matched for age, gender, education level, and hearing thresholds. On-site measurements of ECochG and ABR were taken. The high-density EEG was performed during a routine visit to our hospital.

This study was approved by the Institutional Ethics Review Board of the Shanghai Sixth People's Hospital affiliated with Shanghai Jiao Tong University and was registered in the Chinese Clinical Trial Registry (http://www.chictr.org.cn/index.aspx, registration number: ChiCTR-RPC-17012580). Potential consequences and benefits of the study were explained, and a written informed consent was obtained from every subject before this study.

### Cognitive Test

The MoCA Beijing Version (MoCA-BJ) was administered by professional geriatricians (26), which is considered as an acceptable tool for lower education level groups in both urban and rural areas (27). The MoCA-BJ scale contained seven cognitive domains (5 points-visuospatial and executive function, 3 points-naming, 6 points-attention, 2 points-abstraction, 3 points-language, 5 points-delayed memory, and 6 points-orientation) ranging from 0 to 30, with a higher number indicating better performance. One point was used for education adjustment, in which an additional point can be added to the total score if the individual education years ≤ 12 years.

### ECochG and ABR

The SmartEP auditory evoked potential system (Intelligent Hearing Systems; Miami, FL) was used to measure the ECochG and ABR in a soundproof room. The acoustic stimulation was delivered *via* ER-3A insertable earphones (Etymotic Research; Elk Grove Village, IL). The recording electrode was placed near the tympanic membrane for ECochG or the hairline in the middle of the forehead for ABR, and the reference electrode was on the mastoid. The amplitude and latency of the compound action potential (CAP) in ECochG and waves I and V in ABR were measured in the response to 80 dB HL clicks. The stimulating rate was 13.1 Hz, and the electrical resistance was <3 kΩ. The responses were band-pass filtered between 200 and 2,000 Hz and averaged 1,024 times in each trial.

### Event-Related Potential

#### EEG Acquisition

Electroencephalogram signals were collected in a soundproof room using the Geodesic EEG System (GES 300, Electrical Geodesics; Eugene, OR). A 256-channel HydroCel Geodesic Sensor Net was used to place all the electrodes, and all electrode-skin impedance values were kept below 50 kΩ during the recording. Responses were recorded online relative to a vertex reference electrode (Cz) at a sampling rate of 1,000 Hz and then digitally filtered (0.3–70 Hz). Participants were instructed to keep awake and avoid moving their eyes or changing their posture, and the EEG data were monitored for signs of drowsiness.

#### Event-Related Potential Procedure

**Figure 2:**
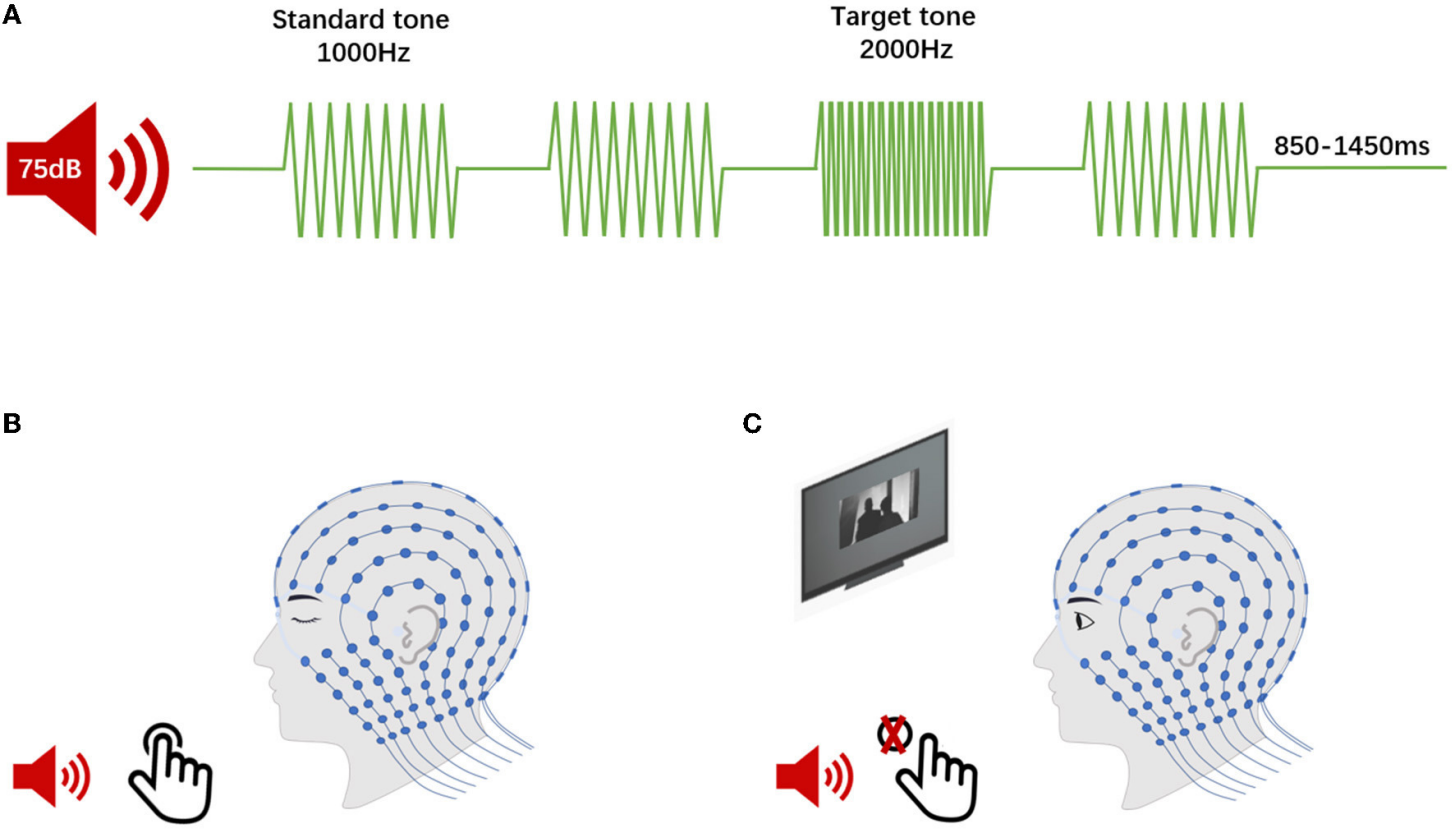
Event-related potentials (ERPs) procedure. **(A)** The continuous auditory stimulus comprised both rarely presented target sounds and frequently presented standard sounds in two tasks. **(B)** The 2-tone auditory oddball task (P300, for top-down analyses). The participants were required to discriminate the target stimulus from the standard tone by pressing a button. **(C)** The passive listening task (MMN, for bottom-up analysis). The participants were instructed to watch the silent movie without responding to the presented auditory stimulus.

The auditory oddball task required participants' responses based on a cognitive decision regarding the auditory stimulus types. The results of this oddball task were interpreted as auditory “top-down” effects, principally (28). Afterwards, in a passive listening task, participants would hear the same stream of auditory stimuli as in the oddball task, and this passive listening task could reflect the “bottom-up” attention effect (28). Therefore, participants engaged in the following two auditory tasks during EEG acquisition (Figure 2): (1) a 2-tone auditory oddball task. The oddball task consisted of two stimuli that were presented in a random order. One stimulus is the quasi-random sequence of frequent standard tones (1,000 Hz, an 85% occurrence probability), while another stimulus is infrequent deviant (target) tones (2,000 Hz, a 15% occurrence probability). The whole task consisted of a total of 1,000 auditory stimuli with random interstimulus intervals (ISIs) ranging from 850 to 1,450 ms. In the oddball paradigm, all stimuli (75-dB sound pressure level with 50-ms duration shaped by a 5-ms rise/fall time window) were delivered through a loudspeaker (Micro-DSP, Sichuan, China) placed 100 cm from the subject at an 180 degrees azimuth. The participants were required to discriminate the target stimulus from the standard tone by pressing a button with their eyes closed to minimize any destructive effects due to alterations in visual attention. (2) A passive listening task used the same series of stimuli in the auditory oddball task. During this task, we showed a silent movie to the participants to divert their attention away from the presented auditory stimuli. They were instructed to watch the movie and not respond to the simultaneously presented target auditory stimuli.

#### ERP Analysis

Event-related potential (ERP) data were analyzed offline with the Net Station 4.3 software (EGI). The continuous EEG signals were digitally filtered between 0.1 and 40 Hz, and then segmented using the event stimulus timestamp. All epochs were calculated 100 ms before and 700 ms after stimulus onset. After segmentation, artifact detection was performed using the Net Station artifact detection tool, which automatically detects eye blinks and eye movements and marks bad channels. Data were baseline-corrected using a 100 ms pre-stimulus period. A single-trial examination was performed for each participant, and artifacts were rejected before grand averages were computed. The P300 elicited by the target in this task is a large, positive-going potential that peaks around 300 ms post-stimulus in normal young adults. The MMN was quantified from the deviant-standard difference waveforms. Peak latency or peak amplitude was determined as the most negative (for MMN) or positive (for P300) point. The amplitude was measured from the baseline, defined as the mean voltage of the pre-stimulus interval, while the latency was measured from the point in time when the deviance occurred (100 ms). We analyzed three (Fz, Cz, and Pz) electrodes to observe the distribution of the P300 and MMN components. Furthermore, the ERP data were input to the GeoSource module of the Net Station software (version 4.5.7) to compute the standardized low-resolution brain electromagnetic tomography (sLORETA) for the purpose of source localization (29, 30).

### Statistics

For parametric data, the results were presented as a mean (SD) or median [interquartile range (IQR)], and for categorical data, as a number (percentage). Depending on the data type, Pearson's 2 test, independent samples *t*-test, and Mann–Whitney *U*-test were used to determine intergroup differences. A linear regression line was fitted to the data to determine the decline rate of cognitive test scores (slope) from 70 to 110 dBA-year of CNE, which was compared using the Mann–Whitney *U*–test. The independent samples *t*-test or the Mann–Whitney *U*–test were used to compare the latencies and amplitudes of AEPs and ERPs between the NG and CG. The 2-tailed *p* < 0.05 was considered to indicate statistical significance, and data analysis was performed using the SPSS 24.0 (IBM, Armonk, NY) and Prism version 9 (GraphPad Software).

## Results

### Baseline Characteristics of Participants

The overall median CNE was ~92.20 dBA-year approximately. In the H-CNE group (*n* = 271), the mean age was 35.2 ± 4.4 years old and the median CNE was 95.2 (92.5–106.4) dBA-year, whereas the mean age of the L-CNE group (*n* = 292) was 33.8 ± 6.4 years and the median CNE was 90.4 (76.0.9–92.2) dBA-year. The subjects in the H-CNE and L-CNE groups were matched well in terms of age, gender, education years, smoking and alcohol drinking habits, and basic diseases. Furthermore, there were no significant differences regarding the terms mentioned above in the same age group ( ≤ 40 years and >40 years) between the H-CNE and L-CNE groups. An overview of the demographic and clinical characteristics is shown in Table 1.

**Table 1:** Demographic characteristics of subjects in the high-cumulative noise exposure (H-CNE) and low-CNE (L-CNE) groups.

	**H-CNE group**			**L-CNE group**			
**Variable**	**≤40 yrs.** **(*n* = 216)**	**>40 yrs.** **(*n* = 55)**	**Overall** **(*n* = 271)**	**≤40 yrs.** **(*n* = 245)**	**>40 yrs.** **(*n* = 47)**	**Overall** **(*n* = 292)**	***P*-value^#^**
Age, mean (±SD), *yrs*.	32.5 ± 4.4	45.7 ± 4.0	35.2 ± 6.8	31.7 ± 4.6	44.5 ± 3.0	33.8 ± 6.4	0.012
Sex, male, (%)	202 (93.5)	51 (92.7)	253 (93.4)	228 (93.1)	41 (87.2)	269 (91.8)	0.483
Education years, mean (±SD), *yrs*.	10.2 ± 2.1	9.4 ± 2.0	10.1 ± 2.1	10.5 ± 2.1	9.8 ± 2.2	10.4 ± 2.1	0.0 76
Exposure duration, mean (±SD), *yrs*.	8.9 ± 4.1***	12.0 ± 5.5**	9.5 ± 4.6	6.6 ± 3.7	8.7 ± 4.3	7.0 ± 4.0	<0.001
CNE, median (IQR), *dBA-year*	94.8 (92.5–105.4)***	96.4 (92.9–106.4)***	95.2 (92.5–106.4)	90.4 (76.0–92.2)	90.1 (77.8–92.2)	90.4 (76.0.9–92.2)	<0.001
Diabetes, *n* (%)	2 (0.9)	2 (3.6)	4 (1.5)	2 (0.8)	0 (0)	2 (0.7)	0.362
Hypertension, *n* (%)	191 (88.4)	43 (78.2)	234 (86.3)	203 (82.9)	38 (80.9)	240 (82.2)	0.176
Smoking, *n* (%)	105 (48.6)	23 (41.8)	128 (47.2)	116 (47.7)	17 (36.2)	133 (45.9)	0.745
Drinking, *n* (%)	96 (44.4)	24 (43.6)	120 (44.3)	103 (42.4)	19 (40.0)	122 (42.1)	0.597
**PTA, mean (±SD)**, ***dB***							
0.25–8 kHz	17.0 ± 4.4***	18.0 ± 4.0	17.16 ± 4.3	15.4 ± 5.0	17.2 ± 4.3	15.67 ± 4.9	<0.001
10–16 kHz	31.2 ± 14.0*	39.2 ± 12.6	32.8 ± 14.1	28.4 ± 13.3	38.7 ± 10.0	30.0 ± 13.4	0.016

# Indicates statistical significance between the H-CNE and L-CNE groups. The number of asterisks indicates statistical significance against the L-CNE in the same age group (*, < 0.05; **, < 0.01; ***, p < 0.001). H-CNE, high cumulative noise exposure group; L-CNE, low cumulative noise exposure group; PTA, pure-tone average (dB HL); yrs, years.

### Cognitive Test Results

**Figure 3:**
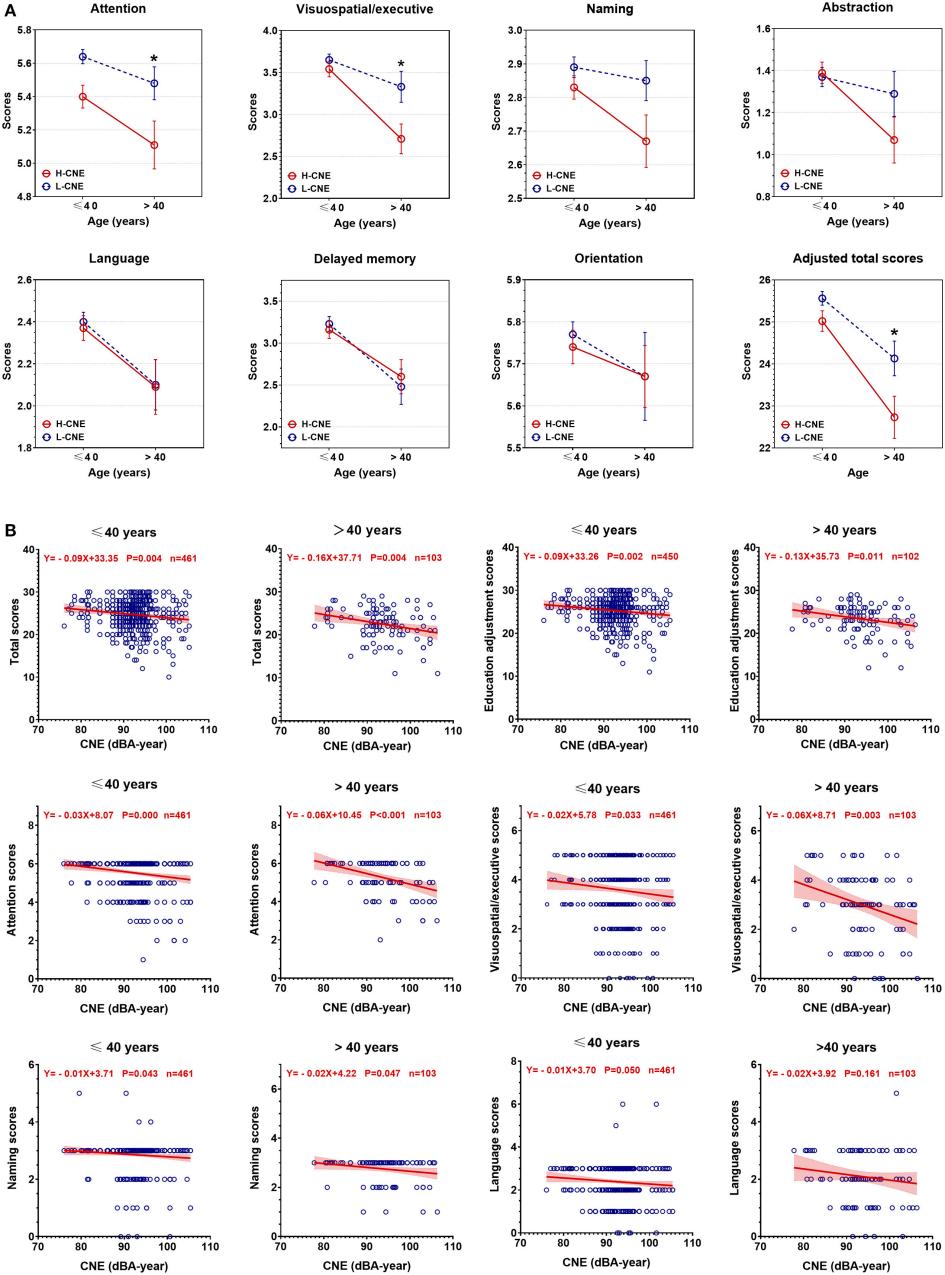
The between-group differences in Montreal Cognitive Assessment Beijing Version (MoCA-BJ) scores. **(A)** Group analysis of MoCA-BJ scores between high-cumulative noise exposure (H-CNE) and low-CNE (L-CNE) groups. For subjects aged under 40 years old, attention function scores were significantly higher in the L-CNE group compared with the H-CNE group. For subjects aged over 40 years old, attention, visuospatial and executive, and education adjustment scores showed a difference between H-CNE and L-CNE. **(B)** The scatter plot depicted the decrease of MoCA-BJ scores with the increase of CNE among participants aged over 40 years or younger. For educational adjusted scores, attention, visuospatial/executive, naming, and language scores, there were significant differences in the rate of decrease in scores with CNE. The asterisks indicates statistical significance between the L-CNE and the H-CNE group in the same age group (*, <0.05; **, <0.01; ***, *p* < 0.001).

Figure 3A presents the results of the MoCA-BJ education adjustment scores and cognitive domain scores in H-CNE and L-CHE subjects. The H-CNE group performed significantly worse than the L-CNE group in the education adjustment scores (24.55 ± 3.71 vs. 25.32 ± 2.62) and domains of attention, visual spatial/executive (5.34 ± 1.02 vs. 5.62 ± 0.67; 3.37 ± 1.37 vs. 3.60 ± 1.13). For subjects under 40 years old, almost all cognitive test scores in the H-CNE group were similar to those in the L-CNE group. Only attention subscales differed significantly between the L-CNE (5.64 ± 0.67) and H-CNE groups (5.40 ± 1.00) (*t* = −3.071, *p* = 0.002). For subjects aged over 40 years, attention scores, visual spatial/executive scores, and education adjustment scores in the H-CNE group were 5.11 ± 1.07, 2.71 ± 1.32, and 22.73 ± 3.72, respectively, while in the L-CNE group, scores were 5.48 ± 0.68, 3.33 ± 1.28, and 24.13±2.83, respectively. There were significant differences in attention scores, visual spatial/executive scores, and education adjustment scores between these two groups (*t* = −2.123, *p* = 0.036; *t* = −2.436, *p* = 0.017; and *t* = −2.436, *p* = 0.017).

Scatterplots revealed a negative relationship between cognitive test scores and CNE, as the values of CNE increased, the corresponding cognitive total scores and subscale scores decreased (Figure 3B). There were significant differences in the rates of decrease in scores among all individuals for educational adjusted scores (*Z* = 1.903, *p* = 0.05), attention scores (*Z* = 2.984, *p* = 0.003), and naming scores (*Z* = 2.131, *p* = 0.033). Among all dimensions of cognitive domains, attention scores were the ones with the fastest decline (slope = −0.03 point/dBA-year, *p* < 0.001 in individuals under 40 years old; slope = −0.06 point/dBA-year, *p* < 0.001 in individuals over 40 years old).

### MMN and P300

Demographic and clinical characteristics of the NG and CG subgroups are compared in Supplementary Table 1. The NG subjects (*n* = 20) were exposed for 8 h/day for an average of 6.9 years, with a mean PTA at 0.25–8 kHz of 9.3 ± 3.1 and 9.8 ± 4.3 dB at 10–16 kHz. Subjects in the CG group (*n* = 20) worked in silent conditions and the mean PTA at 0.25–8 kHz was 10.4 ± 2.7 dB and at 10–16 kHz was 13.1 ± 6.8 dB. There were no significant differences in the amplitude and latency of ABR waves Iand V, as well as the ECochG wave AP between the NG and CG groups (all *p* > 0.05). The other clinical characteristics, such as age, gender, years of education, and cognitive test scores, were not significantly different between the two groups (all *p* > 0.05).

**Figure 4:**
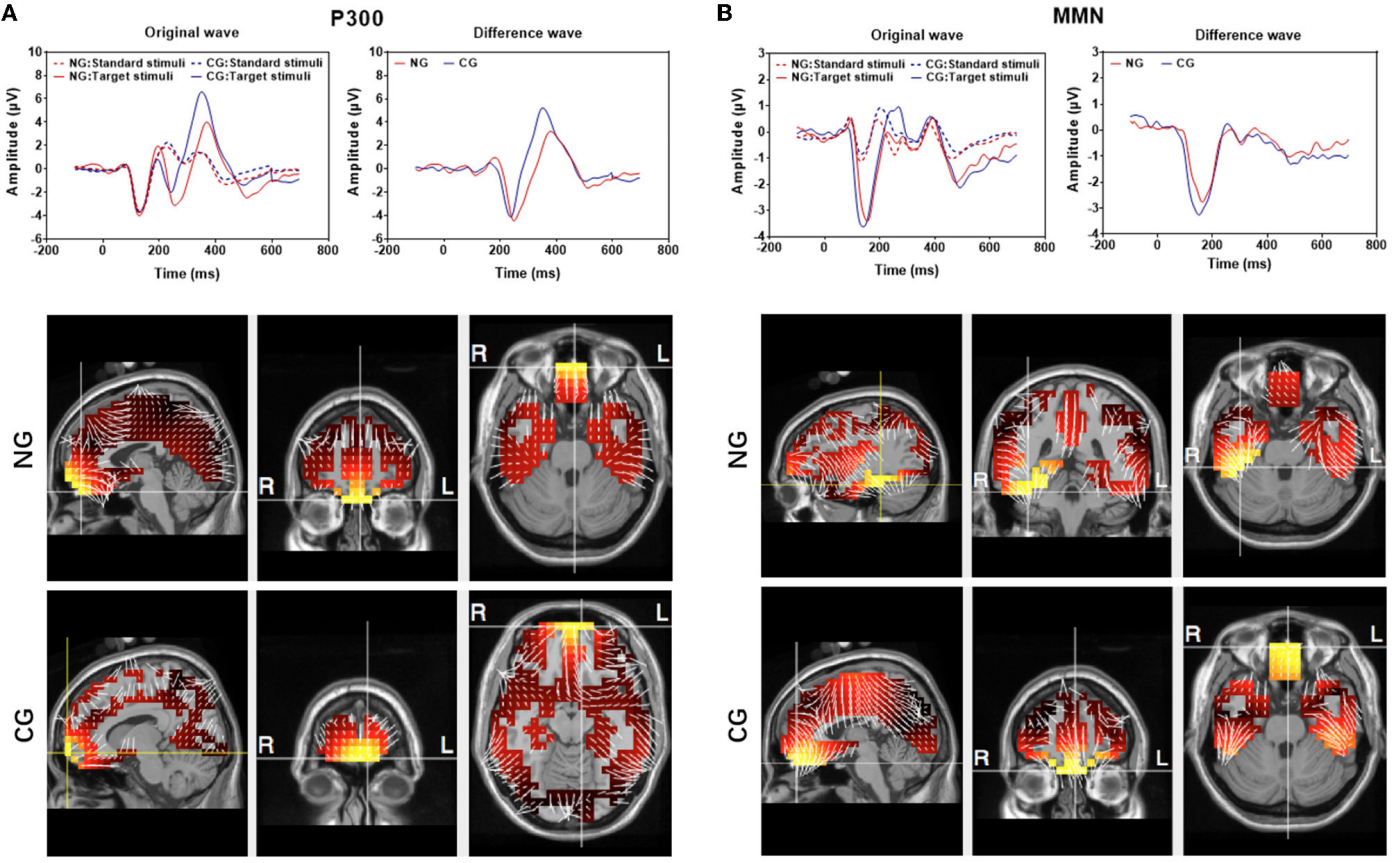
Averaged P300 **(A)** and mismatch negativity (MMN) **(B)** recorded at Cz electrode. Top: Original responses to the standard and deviant stimuli from the 20 subjects in the NG and CG groups. There is a difference between the responses to two types of stimuli. (Dotted lines reflected the response evoked by target stimuli while solid lines reflected the response evoked by standard stimuli; red lines presented the response in NG while blue lines were in CG). Button: sLORETA images of the MMN and P300 components of the two groups at the sagittal, coronal, and axial slices of the maximum current density.

The group-averaged waveforms at Cz are presented in Figure 4 and group-averaged latency and amplitude at Cz, Pz, and Fz are shown in Supplementary Table 2. Overall, deviant stimuli elicited much larger responses from both subgroups in both P300 and MMN measurements. The peak latencies for both P300 and MMN were longer in the responses of NG subjects. In the NG group, subjects' responses had slightly smaller P300 and MMN amplitudes. The P300 latency and amplitude at Cz were 390 ± 12.1 ms and 3.9 ± 2.1 μV, respectively, and the MMN latency and amplitude at Cz were 172.8 ± 15.5 ms and −2.7 ± 0.6 μV. In the CG group, the P300 latency and amplitude at Cz were 369 ± 7.5 ms and 6.7 ± 2.3 μV, respectively, and the MMN latency and amplitude at Cz were 157.8 ± 10.5 ms and −3.2 ± 0.7 μV. The peak latency of MMN from all three sites differed significantly between NG and CG groups (all *p* < 0.01), while there was no significant between-group difference in the amplitudes of MMN (*p* > 0.05).

The source localization was performed in both MMN and P300 by using group-averaged EEG data from the 20 subjects in each group (Figure 4). The maximum current strength of MMN in CG was identified in the front lobe close to the left BA 11 (orbitofrontal area, voxel locations: −3, 52, −27), whereas the maximum current strength of NG was considerably lateralized to the right BA20 (inferior temporal gyrus, voxel locations: 39, −39, −27). The source localization for the maximum current of P300 was in the left BA11, and there was not a significant difference between the NG (locations: −3, 52, −27) and CG (locations: −10, 66, −13) groups.

## Discussion

The present study demonstrated that long-term noise exposure impairs bottom-up and top-down attention functions in the absence of threshold shifts, as evidenced by behavioral and brain responses. The alterations of MMN and P300 suggested impairments in bottom-up and top-down attention functions in participants under long-term noise exposure. In the NG subgroup, significantly lower MMN amplitudes were observed, and the peak latencies of both MMN and P300 were considerably longer. Furthermore, we found a shift of MMN source localization in the right temporal lobe of the noise exposure group, indicating a reorganization of the auditory cortex and alterations of hemisphere dominance. In addition, CNE was a significant factor in the impairment of cognitive function, suggesting that the low-level noise was not as effective compared with high levels of noise.

The association of ambient noise with attention function was less investigated (31, 32), and nearly all early field studies of noise exposure and cognitive performance had some weaknesses, such as small sample sizes, inadequate noise measurement data, and auditory evaluation of each subject accurately. On the other hand, solid evidence from prospective and epidemiological studies (33) revealed that hearing loss was an independent risk factor for cognitive decline, containing the attenuated attention functions, while the mechanism of this association has yet to be elucidated (34). There was likely overlap among the peripheral auditory, central auditory, and cognitive function (35). Animal studies showed that even under a brief exposure to noise, there would be a significant loss of cochlear afferent synapses (36–44). It remained a concern whether such synapse loss could occur in humans and lead to attention function deterioration. Further, noise altered neuronal dendrites (45) and induced peroxidation in specific areas of the lemniscal ascending auditory pathway in mice (46). Noise exposure would result in the substantial impairment of the auditory cortex function and behavioral consequences in mice, regardless of the intensity and duration of noise exposure (47). In the present study, the noise exposure of each subject was documented by their employment duration in the industrial environment, and by the noise survey in the workplaces. All subjects were exposed to industrial noise for 8 h/day for more than 300 days/year. In addition, all individuals maintained good hearing sensitivity over the frequency range from 0.25 to 8 kHz (the hearing thresholds of NG subjects were <25 dB from 0.25 to 16 kHz). The attention deficits observed in this study could be attributable to hard-to-detect cochlea damage and related central plasticity, as there was no interference from hearing threshold or other confounders.

Besides top-down and bottom-up attention, attention could be divided into arousal, sustained attention, selective attention, and divided attention according to hierarchical models from Sohlberg and Mateer (48). Selective attention might be a crucial component of cognitive function (10). The altered amplitude and latency of MMN and P300 could indicate a decrease in not only bottom-up and top-down attention but also selective attention, sustained attention, and divided function (49, 50). On the one hand, the bottom-up and top-down attention models claim that, although distinct processes mediate the attention guidance based on bottom-up and top-down factors, both types of attentional processes require a common neural apparatus, the frontoparietal network (21). On the other hand, the anterior attentional system (AAS), also known as the executive network, oversees selective attention, sustained attention, and divided attention. This system is related to the prefrontal dorsolateral cortex, the orbitofrontal cortex, and the anterior cingulate cortex (48), according to the Posner and Petersen neuroanatomical model (48). The frontoparietal network is clearly the core area of various attention models. Previous animal studies showed that noise exposure could increase oxidative stress, decrease brain-derived neurotrophic factor and synapse-associated protein (51), and cause neuronal dendritic alteration and free radical imbalance in the prefrontal cortex and hippocampus (45). In the present study, we found a significant difference between the NG and CG subgroups in the auditory oddball and the passive listening tasks, indicating a decreased top-down and bottom-up attention process as well as decreased selective, sustained, and divided attention function. In addition, we found that the source localization for maximal MMN was lateralized to the right BA20 (inferior temporal gyrus) in NG subjects, while it was the left BA11 (orbitofrontal area) in CG subjects. These findings were consistent with previous studies, which discovered that the frontal area was the source of MMN in subjects who had not been exposed to noise, and the right temporal lobe appeared to be more susceptible to functional reorganization in subjects who had been exposed to noise (52, 53). Our findings were consistent with that the speech-discrimination-induced ERP was dominant in the right hemisphere in individuals exposed to occupational noise, in contrast to the left hemisphere dominance in control subjects (54). While there was no distinct difference for the P300 source, the underlying mechanisms might be that in noisy environments, bottom-up driven attention is more important during auditory processing (24), and long-term noise exposure might deteriorate bottom-up driven attention function first. Noise exposure induced the reorganization of tonotopic areas (55), as well as structural and molecular changes in human auditory (temporal gyrus) and non-auditory areas (frontal area) (56). However, it was not clear whether similar central plasticity occurs in association with difficult-to-test cochlear damage, which could also reduce the auditory input from cochleae to the auditory brain, although the threshold might not be increased.

Our study has some limitations that should be taken into consideration. We only compare the cognitive performances between different levels of CNE and lack a set of data from the control group of healthy subjects without noise exposure. Our sample size for the EEG measurements remains small, and we cannot completely rule out the existence of peripheral damage in these subjects that requires more sensitive and reliable tests. Due to the large sample size, no further cognitive assessments, such as the Stroop test were performed to evaluate the attention function.

## Conclusions

In conclusion, we found that noise exposure deteriorated both bottom-up and top-down attention functions, as evidenced by the behavioral and brain responses. Behavioral test results revealed that the higher cumulative noise exposure could result in more severe damage to attention function, which was also confirmed by the reduced ERP amplitude and latency. The difficult-to-test cochlear damage, reorganization of auditory and non-auditory areas, and hemisphere dominance alteration might contribute to the significant attention deficits.

## Data Availability Statement

The original contributions presented in the study are included in the article/Supplementary Material, further inquiries can be directed to the corresponding authors.

## Ethics Statement

The studies involving human participants were reviewed and approved by Institutional Ethics Review Board of the Shanghai Sixth People's Hospital affiliated with Shanghai Jiao Tong University. The patients/participants provided their written informed consent to participate in this study.

## Author Contributions

SY and HW: study conception and design. ZJ, HW, JW, and SH: acquisition of data. YW, ZJ, JZ, and YF: analysis and interpretation of data. YW, ZJ, XH, and HW: drafting of manuscript. HW: critical revision. All authors contributed to the article and approved the submitted version.

## Funding

This study was supported by the National Natural Science Foundation of China (82071041/H1304), Innovative research team of high-level local universities in Shanghai (SHSMU-ZLCX20211702), Young Scientists Fund of the National Natural Science Foundation of China (Grant No. 82101220), the First Grant (2020YFC2005201) of Chinese National Key Research and Development Program (2020YFC2005200).

## Conflict of Interest

The authors declare that the research was conducted in the absence of any commercial or financial relationships that could be construed as a potential conflict of interest.

## Publisher's Note

All claims expressed in this article are solely those of the authors and do not necessarily represent those of their affiliated organizations, or those of the publisher, the editors and the reviewers. Any product that may be evaluated in this article, or claim that may be made by its manufacturer, is not guaranteed or endorsed by the publisher.
