# Supplementary material for: Bottom-Up and Top-Down Attention Impairment Induced by Long-Term Exposure to Noise in the Absence of Threshold Shifts
Source: Front Neurol. 2022 Mar 1;13:836683. doi: 10.3389/fneur.2022.836683 (PMC8920971; doi:10.3389/fneur.2022.836683)
Supplement: Supplementary file 1 [file Table_1.pdf]

Supplementary Table 1. Demographic and hearing characteristics of subjects in the NG and CG subgroups.

Variable	**NG (20)**	**CG (20)**	P-value
Characteristic			
Age, mean (±SD), yrs.	29.3±3.8	27.7±3.6	>0.05
Male, n, (%)	18 (90)	18 (90)	>0.05
CNE, mean (±SD), dBA-year	83.6±2.8	NA	NA
Exposure duration, mean (±SD), yrs.	6.9±1.9	NA	NA
Education level, mean (±SD), yrs.	11.6±1.4	12.4±1.2	>0.05
PTA, mean (±SD), dB			
0.25-8 kHz	9.3±3.1	10.4±2.7	<0.001
10-16 kHz	9.8±4.3	13.1±6.8	<0.001
ECochG-CAP			
Latency, mean (±SD), ms	1.6±0.2	1.7±0.1	>0.05
Amplitude, mean (±SD), μV	0.4±0.1	0.4±0.1	>0.05
ABR Wave I			
Latency, mean (±SD), ms	1.5±0.1	1.5±0.1	>0.05
Amplitude mean (±SD), μV	0.3±0.1	0.3±0.1	>0.05
ABR Wave V			
Latency (ms), mean (±SD), ms	5.6±0.2	5.5±0.2	>0.05
Amplitude mean (±SD), μV	0.5±0.1	0.5±0.1	>0.05

NG: noise group with a hearing threshold ⩽25dB HL across the frequency range 0.25-16 kHz;

CG: control group without noise exposure history. PTA: pure-tone average (dB HL). yrs: years. NA: not applicable.

Supplementary Table 2. The peak latencies and amplitudes of MMN and P3 in the NG and CG groups.

	**NG** (20)		**CG** (20)	
	Latency(*ms*)	Amplitudes(μV)	Latency(*ms*)	Amplitudes(μV)
**MMN**				
Cz	172.8±15.5***	-2.7±0.6	157.8±10.5	-3.2±0.7
Fz	169.2±13.5**	-2.9±1.2	148.4±13.5	-3.6±0.8
Pz	147.2±18.1***	-2.5±0.7	132.1±12.4	-2.6 ± 0.7
**P300**				
Cz	390.7±12.1***	3.9±2.1***	369.4±7.5	6.7±2.3
Fz	367.0±9.4***	1.2±0.7***	349.7±9.4	5.3±1.4
Pz	369.5±21.5**	4.3±1.6***	352.6±9.7	6.4±2.0

The number of asterisks indicates statistical significance against the CG (**: $p < 0.01$, ***: $p < 0.001$).
